# Behavioral health coaching for rural veterans with diabetes and depression: a patient randomized effectiveness implementation trial

**DOI:** 10.1186/1472-6963-14-191

**Published:** 2014-04-28

**Authors:** Jeffrey A Cully, Jessica Y Breland, Suzanne Robertson, Anne E Utech, Natalie Hundt, Mark E Kunik, Nancy J Petersen, Nicholas Masozera, Radha Rao, Aanand D Naik

**Affiliations:** 1 Houston VA HSR&D Center for Innovations in Quality, Effectiveness and Safety, (MEDVAMC 152), 2002 Holcombe Blvd., Houston, TX 77030, USA; 2 Michael E. DeBakey VA Medical Center, 2002 Holcombe Blvd., Houston, TX 77030, USA; 3 Baylor College of Medicine, Houston, TX, USA; 4 VA South Central Mental Illness, Education, Research and Clinical Center, Virginia, USA

**Keywords:** Behavioral medicine, Diabetes mellitus, Depression, Veterans’ health

## Abstract

**Background:**

Depression and diabetes cause significant burden for patients and the healthcare system and, when co-occurring, result in poorer self-care behaviors and worse glycemic control than for either condition alone. However, the clinical management of these comorbid conditions is complicated by a host of patient, provider, and system-level barriers that are especially problematic for patients in rural locations. Patient-centered medical homes provide an opportunity to integrate mental and physical health care to address the multifaceted needs of complex comorbid conditions. Presently, there is a need to not only develop robust clinical interventions for complex medically ill patients but also to find feasible ways to embed these interventions into the frontlines of existing primary care practices.

**Methods/design:**

This randomized controlled trial uses a hybrid effectiveness-implementation design to evaluate the Healthy Outcomes through Patient Empowerment (HOPE) intervention, which seeks to simultaneously address diabetes and depression for rural veterans in Southeast Texas. A total of 242 Veterans with uncontrolled diabetes and comorbid symptoms of depression will be recruited and randomized to either the HOPE intervention or to a usual-care arm. Participants will be evaluated on a host of diabetes and depression-related measures at baseline and 6- and 12-month follow-up. The trial has two primary goals: 1) to examine the effectiveness of the intervention on both physical (diabetes) and emotional health (depression) outcomes and 2) to simultaneously pilot test a multifaceted implementation strategy designed to increase fidelity and utilization of the intervention by coaches interfacing within the primary care setting.

**Discussion:**

This ongoing blended effectiveness-implementation design holds the potential to advance the science and practice of caring for complex medically ill patients within the constraints of a busy patient-centered medical home.

**Trial registration:**

Behavioral Activation Therapy for Rural Veterans with Diabetes and Depression: NCT01572389.

## Background

Depression and diabetes cause significant burden for patients and the healthcare system [1,2]. Individuals with diabetes have up to a 24% increased risk of developing depression [3], and individuals with depression have increased risk of developing type 2 diabetes [4]. When co-occurring with diabetes, depression is associated with poorer self-care behaviors and worse glycemic control than in diabetes without depression [5]. Unfortunately, the clinical management of co-occurring diabetes and depression is complicated by a host of patient, provider, and system factors that, ultimately, lead to limited opportunities for comprehensive integrated healthcare treatments, especially for patients in rural locations [6,7].

Effective interventions exist for depression [8] and diabetes [9], but these interventions often have limited impact when depression and diabetes co-occur [10,11]. There is currently a need to develop interventions that simultaneously target diabetes and depression [11], with several recent studies showing promising preliminary results [12,13]. Blended diabetes and depression interventions offer the potential to improve broad patient outcomes but also offer opportunities to integrate physical and mental health care within interprofessional team-based healthcare settings, [13,14].

Unfortunately, the limited availability of specialized mental and behavioral health providers is a significant barrier to integrated health care in primary care settings and is especially problematic for rural patients [15]. In response, researchers and clinicians have tried to increase the availability of providers and behavioral health services. For example, training non-mental health clinicians to provide basic mental health services has been shown to improve the provision of mental health care in primary care settings (e.g., Areán et al. [16]). Other modifications include the use of telephone-based mental health care, which eases travel burden on patients, and, in some contexts, appears to perform as well as face-to-face care, with lower attrition rates [17]. These techniques may be especially important for rural populations, which have higher rates of diabetes than urban populations [18] and limited access to both diabetes and mental health care [19,20].

From a healthcare-system perspective, the patient-centered medical home (PCMH), a primary focus of healthcare reform in the United States, offers opportunities for improved access to integrated and coordinated diabetes and depression care. Unfortunately, a dearth of research has assessed coordinated or blended models of diabetes and depression care in PCMHs. In 2010, the Veteran’s Health Administration began implementing a PCMH model known as the Patient-Aligned Care Team (PACT). PACTs are situated in primary care clinics and are intended to improve the provision of patient-centered care by placing the patient at the center of a care team that includes a primary care provider, a registered nurse, a licensed vocational nurse or health technician, and a clerical associate. PACTs are responsible for the coordination of patient healthcare services and represent an ideal setting for the integration of mental and physical healthcare practices.

The current article describes a Veterans’ Affairs (VA) Health Services Research & Development-funded multiclinic, patient-level randomized controlled trial designed to examine clinical effectiveness and preliminary implementation outcomes of a blended depression and diabetes behavioral health coaching intervention. Delivered over the telephone by PACT clinicians and trained behavioral health coaches, the intervention is intended for rural Veterans with uncontrolled diabetes and clinically elevated symptoms of depression. The trial has two primary goals: 1) to examine the effectiveness of the intervention on both physical (diabetes) and emotional health (depression) outcomes and 2) to simultaneously pilot test a multifaceted implementation strategy designed to increase fidelity and utilization of the intervention by coaches interfacing within the primary care setting. This simultaneous focus on effectiveness and implementation outcomes, defined by Curran et al. [21] as a hybrid effectiveness-implementation trial, is intended to reduce the translation lag from intervention development to intervention implementation and adoption in routine practice. However, hybrid designs require a balance between the need for internal control (e.g., scientific manipulation) and external validity (e.g., ability of the study to generalize to the intended clinical care setting). This article outlines such a hybrid design, including decisional points related to the study team’s desire to explore patient-, clinician-, and system-level outcomes of a blended diabetes and depression coaching intervention.

## Methods

The Healthy Outcomes through Patient Empowerment (HOPE) study is being conducted at the Michael E. DeBakey Veterans Affairs Medical Center (MEDVAMC) and six affiliated community-based outpatient clinics (CBOCs) in Southeast Texas. The study is approved by the Baylor College of Medicine Institutional Review Board and the MEDVAMC Research and Development Committee.

**Figure 1:**
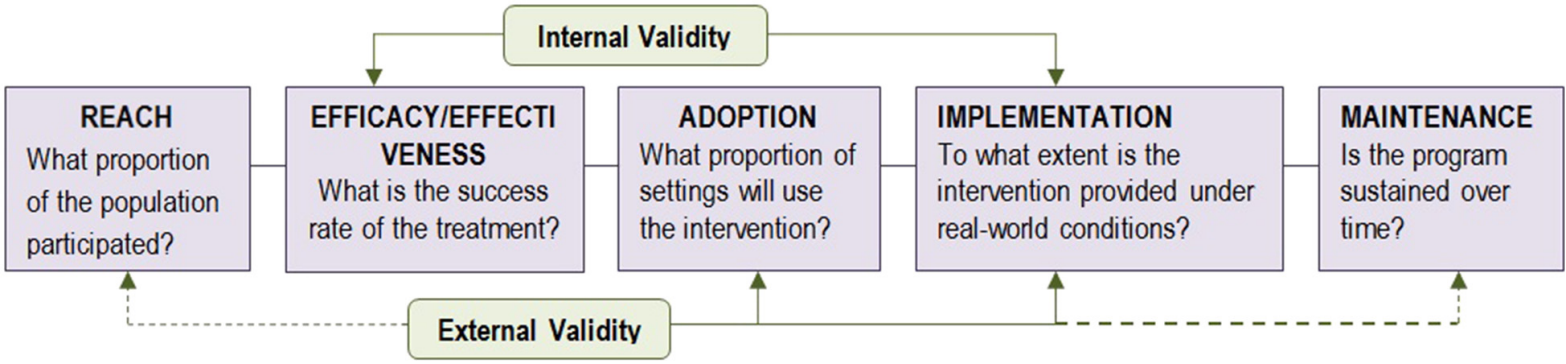
Re-aim evaluation framework.

Using a hybrid effectiveness-implementation design, the project relies heavily on the RE-AIM evaluation framework (Reach, Effectiveness, Adoption, Implementation, Maintenance) [22,23] (see Figure 1). Although we discuss the effectiveness and implementation aspects separately in this article, the application and evaluation of procedures within hybrid trials of this nature occur simultaneously and are often contingent upon one another. For example, the eventual success of the clinical intervention will be impacted by the success (or failure) of the implementation strategy and the ability of coaches to effectively deliver the intervention.

### Clinical effectiveness

#### Identification and recruitment of participants

The study will enroll 242 participants with uncontrolled diabetes (i.e., average HbA1c values ≥ 7.5% for the last year) and coexistent, clinically elevated symptoms of depression (i.e., the nine-item Patient Health Questionnaire [PHQ-9] score ≥ 10). Using an unequal randomization design, we will randomize 60% of participants (n = 145) to receive blended diabetes/depression behavioral health coaching for six months (active intervention), followed by six months without coaching (maintenance period); the other 40% (n = 97) will receive enhanced usual care (EUC), which includes the provision of educational materials. Primary care team members (i.e., the primary care physician, registered nurse, licensed vocational nurse) of each consented participant who completes the baseline interview will be informed that the Veteran endorsed mild, moderate or severe symptoms of depression and has consented to participate in a study related to the management of diabetes and depression. Notification will occur through the electronic medical record (EMR) system and secure messaging platforms.

Potential participants are being identified through VA databases pulled from the Veterans Integrated Service Network for Southeast Texas 16 data warehouse. These data provide the study team with a list of patients with primary care appointments within the last 24 months and an International Classification of Diseases, Ninth Revision code for diabetes (250.××). The study’s data analyst runs the list through an analytic algorithm for geographic exclusion (i.e., patients must reside at least 20 miles from the MEDVAMC and/or receive primary care at a CBOC, to ensure rural status) and uncontrolled glucose (i.e., average A1C levels ≥ 7.5% for at least one year). Eligibility criteria for the study are designed to be as broad as possible to closely resemble the targeted patient population and to provide the greatest external validity for the intervention. Patients are excluded only for factors that would make a telephone-based coaching intervention delivered by primary care providers inappropriate (e.g., active suicidal ideation; severe cognitive impairment; severe mental health condition, such as psychosis or active substance-abuse disorder; significant vision or hearing loss). Research assistants conduct chart reviews to confirm glucose-control status and geographic location, as well as to exclude individuals with psychotic disorders, cognitive impairment, or active substance-abuse issues. For safety, the chart review is also used to exclude patients with a history of significant hypoglycemic events. Remaining patients are sent an opt-out letter and directed to call a hotline number if they are not interested in participating.

Calls are made to all potential participants who do not opt-out. Interested participants complete a telephone-based screening interview with a research assistant to confirm eligibility, based on a brief screening for depression and to assess for factors that would preclude engaging in the telephone-based intervention (e.g., hearing loss, lack of regular access to a telephone, relocation outside Southeast Texas). We also exclude patients who do not endorse symptoms of depression on a brief screening for depression using the PHQ [24,25] during the screen appointment. Patients endorsing active suicidal ideation and requiring immediate mental health attention at any point in the recruitment process are referred for appropriate services and excluded from the study.

**Figure 2:**
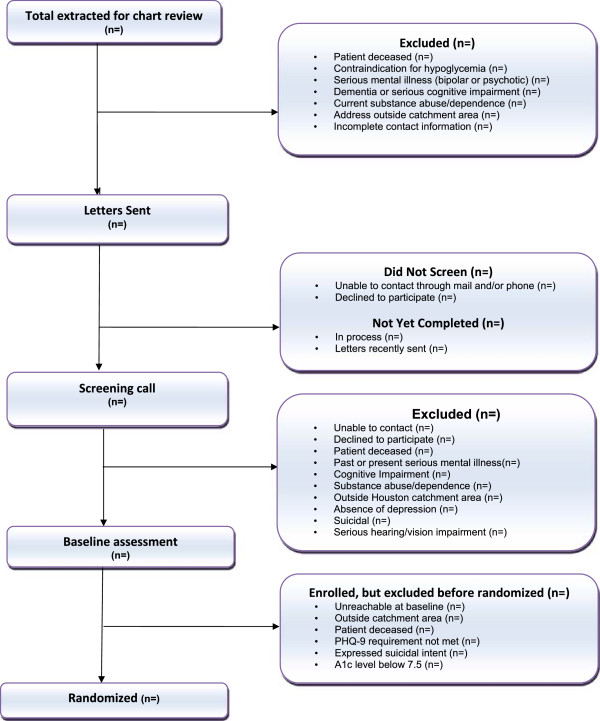
Consort diagram.

Eligible participants who provide informed consent complete the baseline telephone assessment. Participants scoring < 10 on the PHQ-9 at the baseline assessment are excluded from further participation in the study. Participants meeting the inclusion criterion for depression status at baseline are then asked to confirm their eligibility on the glycemic-control criterion with a baseline blood draw. Participants with A1C levels < 7.5% are excluded. Upon final determination of inclusion, participants are randomized to HOPE or EUC. Participants are compensated only for research assessments: $30 for participation in baseline, six- and 12-month assessments (total of $90). Additional information on participant identification and recruitment is presented in the form of a CONSORT diagram (see Figure 2).

#### Clinical intervention

HOPE delivers six biweekly (twice a month) 30-minute sessions and three monthly 15-minute follow-up sessions over a six-month period. All sessions are completed over the telephone to ease treatment burden on participants. Participants receive two “core” sessions that provide foundational information and activities related to diabetes, depression, and setting effective goals and action plans. During the core sessions, participants also select skill-based activities from the intervention manual (e.g., modules) that most closely align to their interests and most pressing needs (see Table 1). These “elective modules” are provided during sessions three through six. The final three sessions (seven through nine) provide opportunities for solidification of skills and maintenance of lifestyle modifications. A detailed description of the HOPE intervention, along with pilot data from a small open trial, can be found in a previously published study [12].

**Table 1:** HOPE intervention modules

Session 1	**Introduction to HOPE**
	• Introduction to HOPE and how it can help
	• Rapport Building
	• Introduction to behavioral activation
	• Setting behavioral activation goal
Session 2	**Setting goals and making action plans**
	• Introduction to principles of goal setting and action planning
	• Introduction/choice of skills areas
Sessions 3–6 (Skill sessions chosen by participants)	**Increasing pleasant activities**
	• Connections between activities and mood
	• Benefits of increasing pleasant and meaningful activities
	• Setting goal and action plan to increase pleasant activities
	**Using thoughts to improve wellness**
	• Connections between thoughts and mood
	• Recognizing and reducing unhelpful thinking
	• Setting goal and action plan to use thoughts to improve mood
	**Eating wisely**
	• Information about healthy eating
	• Setting goals and action plans to improve diet:
	◦ Limiting portions
	◦ Controlling carbohydrates
	◦ Increasing fruit/vegetable consumptions
	◦ Reducing unhealthy fat intake
	**Being physically active**
	• Benefits of physical activity
	• Setting goals and action plans to increase activity
	**Managing your medications**
	• Importance of taking medications as prescribed
	• Setting goals and action plans to increase activity related to:
	◦ Knowing medications
	◦ Choosing the right diabetes medication
	◦ Keeping a schedule for taking medications
	◦ Medications for mood
	**Learning how to relax**
	• Relationships between stress, anxiety and worry
	• Setting goals and action plans to increase activity related to:
	◦ Deep breathing to reduce stress and tensions
	◦ Imagery to reduce stress and tension
Sessions 7–9 (follow-up)	**Adjusting action plans and overcoming obstacles**
	• Checking on progress and resolving barriers to goal attainment

The HOPE intervention is based on principles of goal setting and action planning [26-28] and evidence-based psychotherapy, including cognitive-behavioral therapy [29,30] and motivational interviewing [31,32]. As such, HOPE emphasizes the relationship between the coach and patient as a necessary element for change and leverages that relationship to implement focused skill-based techniques designed to help patients improve their physical and/or emotional self-management behaviors. Sessions are based on the concept of patient self-management and the development of physical and emotional health skills to address both diabetes and depression. The primary skill-based technique is that of behavioral activation, which encourages patients to set focused behavior-oriented goals and action plans to address their physical and emotional health concerns. Diabetes skill management focuses on diabetes awareness and the role of diet, exercise, and medication management in improving diabetes outcomes. Depression skill management is based on traditional cognitive and behavioral strategies, such as identification of maladaptive thinking, behavioral activation, and relaxation. Notably, the diabetes and depression intervention skill “modules” are viewed as overlapping, with the potential to improve the management of either depression or diabetes (e.g., depression may be improved by exercise or diet skills, whereas diabetes may be improved by behavioral activation).

#### Intervention providers – “coaches”

The study enlists the participation of coaches embedded in primary care, who deliver the HOPE intervention and subsequently communicate with each participant's primary care team through the EMR, secure (instant) messaging, and telephone contacts. Such contacts focus on communicating the participant’s ongoing behavioral health-improvement goals and addressing any notable physical or emotional health changes that might require further intervention (e.g., hypoglycemia, medication issues, or intent or plan for self-harm). See the Implementation Section for additional details.

#### Outcome measures, analyses, and power

Clinical effectiveness is being measured with depression scores on the PHQ-9 [33] and HbA1c values at posttreatment (six months) and at a 12-month follow-up. To minimize potential bias, all self-report measures will be collected by blinded independent evaluators.

Additional data are collected at six- and 12-month follow-up assessments, including the following: the a) Problem Areas in Diabetes Questionnaire [34] to assess changes in diabetes distress; b) Penn State Worry Questionnaire [35] to assess changes in worry/anxiety; and c) Goal-Setting Evaluation Tool for Diabetes [36] to assess goal-setting quality. Other study variables include demographic variables, healthcare use, self-efficacy, comorbid physical and mental conditions and other psychological factors.

All analyses will be done on an intention-to-treat basis. Hierarchical linear models will be used to assess differences in outcomes measures between baseline and six- and 12-month follow-ups. Effect sizes will be compared between treatment and control groups at those time points.

Unequal randomization will be used, with 60% of patients randomized to the treatment group and 40% randomized to EUC. A final cohort of 182 participants (109 in the active intervention arm and 73 in the control arm) at 12-month follow-up (i.e., recruitment of 242 to allow for 25% attrition) provides 80% power to detect an effect size of 0.45. This effect size represents a clinically meaningful difference in HbA1c of 0.5 (*SD =* 1.1) [37]. For the PHQ-9, a reduction of 5 points or greater will be considered clinically significant [38-40]). Our sample size of 109 intervention participants and 73 controls will have 80% power to detect a change as small as 1.5 points in the PHQ-9 score with a two-sided alpha = 0.05.

### Implementation

As a hybrid type 2 trial [21], this study assesses effectiveness and implementation outcomes simultaneously to ensure that, if effective, the HOPE intervention will be well positioned for use within existing primary care settings. As a second aim, the current trial seeks to examine and pilot a collection of training- and facilitation-based interventions designed to enhance the adoption and implementation of the HOPE intervention by frontline primary care clinicians. Targeted care providers include nurses, social workers, pharmacists, registered dietitians, psychologists, and physician assistants.

The RE-AIM framework was used to define salient study constructs and to guide evaluation of the implementation strategy, which is focused on Adoption and Implementation [22]. For the purposes of this study, adoption refers to the percent and representativeness of clinic staff and settings using the intervention relative to the total number of clinics and clinicians approached. Although broad adoption statistics are being collected, additional details, including barriers and facilitators to using the intervention, are also being collected, using survey and qualitative interview methods. Implementation, as defined by RE-AIM, refers to the consistency or fidelity in delivering the intervention across staff and settings. Fidelity is being measured using audio taped session reviews as part of the coaching audit and feedback procedures (see below).

#### Coaches

The intervention is being delivered by clinician health coaches with varying professional backgrounds (e.g., nurses, clinical social workers, dieticians, etc.). Given the broader effectiveness goals of the study, interventionists include “study coaches” and “PACT coaches.” Study coaches are defined as clinicians providing care as part of their duties to the study project itself. Study coaches provide care outside the physical setting of primary care, are not formal members of the PACT, and communicate with the PACT exclusively at a distance through the EMR, instant messaging, and telephone. Study coaches are included in the project to simulate opportunities to reach patients using care providers who are not directly associated with the PACT (e.g., telephone-based care-management services that are not physically located in the primary care treatment setting). Nonprimary care-based coaches may improve access to comprehensive healthcare services for patients in rural and underserved geographic locations where resources may be limited. For the purposes of this investigation, it is anticipated that study coaches will include an interprofessional mix of professionals, such as masters-level providers and trainees from psychology, social work and pharmacy. Study coaches will be identified by the project team through outreach to non-primary care programs, such as hospital-based specialty care service organizations and training programs. Notably, study coaches are viewed as distinct from PACT coaches due to their physical presence outside the primary care setting.

PACT coaches, in contrast to study coaches, are formal members of the PACT and assimilate the HOPE intervention into their regular primary care clinical duties. PACT coaches are clinicians who are able to deliver telephone-based coaching within their scope of professional practice. Future implementation of PACT coaches will occur through the training of existing clinicians within the primary care setting.

#### Implementation strategy

Several implementation techniques are being used to support coaches as they attempt to adopt and effectively use the HOPE intervention. Implementation techniques are multifaceted and target both the ability of coaches to accurately and effectively use the content and procedures of the intervention, as well as provide support for coaches in their ability to incorporate the intervention into the primary care setting. Initial training and facilitation, developed in collaboration with an advisory council consisting of frontline PACT clinicians, occur through a focused coach training workshop and a subsequent pairing of coaches with HOPE mentors. Mentors are members of the study team who have behavioral-change expertise. In addition, mentors are educators with skills in helping others with professional development. Mentors use audit and feedback of coach-session audiotapes as a foundation for coach professional development. A third approach relates to the study’s goal to disseminate intervention efforts to PACT providers and the larger PACT through the use of structured medical-record note templates designed to embed treatment progress notes within each patient’s EMR, regular facilitation calls with coaches to share experiences across intervention sites, and regular HOPE newsletters delivered to a broad audience of primary care clinicians and leaders.

##### Initial coach training

The initial coach training occurs over two, two-hour computer-aided telephone sessions. The distance-based learning format improves feasibility and has the added benefit of modeling effective telephone communication, a necessary skill for coaches providing a telephone-based treatment. The first coach training session covers background information on diabetes and depression, the objectives and core components of HOPE, including HOPE’s approach to patient care; the coaching relationship; and evidence-based techniques to treat diabetes and depression, such as motivational interviewing, behavioral activation, and goal setting. The second coach training session reviews specific details of treatment sessions and describes how to apply the principles learned during the first training session. Each training session uses a variety of interactive elements to provide coaches with the practical elements of using HOPE, such as computer-aided demonstrations of techniques, audio recordings and discussions of simulated patient sessions. Telephone trainings are supplemented with materials provided to coaches through the study website, including a treatment manual, patient workbook, and optional concept reviews on diabetes and depression.

##### Mentoring with audit and feedback

HOPE mentors augment the telephone training and facilitate advanced levels of practice through the use of audit and feedback procedures. All coaches audio record treatment sessions with HOPE participants. Mentors rate sessions using a clinician fidelity form, developed from a prior study [41]. Mentors listen to all sessions for each coach’s first patient and review a random set of sessions on a quarterly basis for all subsequent patients. Coaches remain in contact with mentors throughout the course of the trial to discuss clinical cases by email or telephone.

##### Facilitation

On a monthly basis, coaches have the option of participating in a conference call with other HOPE coaches, mentors, and members of the study staff (e.g., principal investigators, research coordinators). These calls are intended to help coaches overcome practice and organizational barriers to the implementation of the HOPE intervention. HOPE staff organize and administer the call; however, the needs and interests of the coaches largely dictate the agenda, so as to increase coach-to-coach interactions and sharing. To accommodate coaches’ schedules, the calls are optional, with the frequency of calls adjusted according to coach preference. Minutes from the meeting are posted on the HOPE website, with summaries provided in the HOPE newsletter for coaches unable to attend.

#### Outcome measures and analyses

Implementation analytics are largely descriptive in nature. The RE-AIM facet of Adoption will be measured as the percent and representativeness of clinic staff and settings using the intervention relative to the total number of clinics and clinicians approached. The RE-AIM facet of Implementation will be assessed through formal fidelity ratings provided by mentors.

## Discussion

HOPE is a patient-level randomized controlled trial designed to streamline and improve the transition from research to practice by simultaneously examining the clinical effectiveness and implementation potential of a telephone-based approach to blended diabetes/depression care for the primary care setting. From an effectiveness standpoint, the blended diabetes and depression focus, along with the multifaceted patient-centered approach of the intervention, represent novel clinical enhancements. The study also employs a comprehensive implementation strategy to support the use of the intervention within the primary care setting, provides additional innovation both in terms of opportunities to enhance treatment effectiveness (e.g., improved communication between interventionists and the primary care treatment team) and in terms of providing opportunities to better understand the potential for frontline clinicians to effectively use the HOPE program within their existing patient population. It is anticipated that a focus on implementation will facilitate a better understanding of the challenges and opportunities for future dissemination of this blended intervention approach should the project produce significant positive clinical outcomes. Notably, project implementation efforts have been closely aligned with current VA clinical practice and policies. For example, services obtained as part of this intervention are consistent with healthcare coverage policies for patients receiving care through the VA.

The HOPE clinical intervention differs from prior work on diabetes and depression in three distinct ways. First, the intervention allows patients to direct the focus of treatment on diabetes or depression, or both, and flexibly attends to the most pressing needs of patients. Thus, it is not a stepped-care approach led by clinicians but rather a patient-centered approach to the management of diabetes and/or depression. Research suggests that patients want to be involved in care decisions [42], and patients’ involvement in choosing treatment content is a foundational feature of HOPE. The intervention engages patients to actively select and direct care practices, hypothesized to increase patient motivation and confidence to achieve self-initiated goals.

Second, the clinical intervention is conducted entirely over the telephone. A recent review suggests that telehealth services, including video-based services, hold the potential to improve access to mental and physical health care [43,44]. However, prior studies conducted within the VA suggest that the effectiveness of telephone-based depression treatment with Veterans may produce limited treatment effects [45]. Additional research studies on programs that provide telemental health treatment, like HOPE, are needed.

As a third level of innovation, the clinical intervention, as well as facets of the implementation strategy, seek to increase the overall potency of outcomes through directed efforts to communicate clinical information, e.g., presence of clinically significant depressive symptoms in medically ill patients, to each patient’s primary care treatment team. The inclusion of the patient’s existing provider team is seen as critical, not only for improving intervention potency but also for reconciling discrepant definitions of treatment adherence and health-related goals between patients and providers, and avoiding potential clinical pitfalls related to hypoglycemia and worsening emotional health.

From an implementation standpoint, the project enlists coach-based “interventions” to increase the fidelity and adoption of HOPE by PACT providers with varying levels of clinical experience. The training of nonmental health experts is viewed as an important step to address the limited supply of such providers in primary care and in clinical settings outside academic medical centers. To adequately train and support these nonmental health specialists, the project team collaborated with stakeholders to create print and online training materials, a focused four-hour training workshop for clinician coaches, and a comprehensive mentor program to enhance the professional development and clinical skills of HOPE coaches.

As others have noted, strategies to improve access to care must be balanced with the need to retain intervention fidelity and effectiveness to keep these innovative treatments from being “brilliant, but irrelevant” [46]. To this end, HOPE attempts to balance the need to document the effectiveness of the clinical intervention while maintaining and supporting the use of the treatment in real-world primary care settings.

Although not a primary or secondary outcome for the trial, the project will explore clinician factors associated with treatment fidelity, including clinician professional background, prior diabetes treatment experience, prior depression treatment experience, and time dedicated to primary care relative to other professional duties. Further, the inclusion of study coaches will allow an exploration of data to determine whether HOPE is more effective when provided by the study coaches, who operate outside the fast-paced, competing-demand setting of primary care. It is anticipated that study coaches will have flexible schedules and greater ability to engage patients in a timely manner, given their project funding and distance from primary care. However, because study coaches are physically distant from the primary care teams, PACT coaches may evidence more frequent and higher-quality communication with other clinicians within the PACT, thus increasing the potency and patient-centeredness of the intervention.

Because of the multifaceted nature of the trial, it is anticipated that the HOPE intervention will provide meaningful clinical information for the treatment of complex patients with diabetes and depression. This trial will also supply critical implementation data for the potential use of telephone-based coaching interventions in the primary care setting. It is believed that hybrid effectiveness-implementation designs, although complex and not without sacrifices to internal validity, are useful for not only reducing lag time between efficacy and implementation but also for providing opportunities to identify potential pitfalls, including information to better understand a negative clinical trial.

## Abbreviations

PCMH: Patient-centered medical home; PACT: Patient-aligned care team; VA: Veterans Affairs; HOPE: Healthy Outcomes through Patient Empowerment; MEDVAMC: Michael E. DeBakey VA Medical Center; CBOC: Community-based outpatient clinic; RE-AIM: Reach Effectiveness, Adoption, Implementation, Maintenance; PHQ: Patient Health Questionnaire; EUC: Enhanced usual care; EMR: Electronic medical record.

## Competing interests

The study was funded by a grant (IIR #10-135) from the VA HSR&D Research Service Line. No other competing interests are known.

## Authors’ contributions

JAC: multiple principal investigator, conceptualized overall study design, obtained grant funding, codeveloped the clinical and implementation interventions, provided critical revisions of the manuscript, and approved final version. JYB: drafted the initial version of the manuscript, provided critical revisions, and approved final version. SR: assisted with training of intervention coaches and provided critical revisions to the manuscript. AEU: assisted with the project implementation model and provided critical revisions to the manuscript. NH: assisted with the implementation model and training of intervention coaches and provided critical revisions to the manuscript. MEK: conceptualized overall study design, assisted with grant development and submission, and provided critical revisions to the manuscript. NJP: provided critical revisions to the manuscript, developed study analytical model and participated in grant development. NM: provided critical revisions to the manuscript and participated in grant development. RR: provided critical revisions to the manuscript and participated in grant development. ADN: multiple principal investigator, codeveloped the clinical and implementation interventions, conceptualized overall study, obtained grant funding, provided critical revisions of the manuscript, and approved final version. All authors read and approved the final manuscript.

## Pre-publication history

The pre-publication history for this paper can be accessed here:

http://www.biomedcentral.com/1472-6963/14/191/prepub
